# Electronic medical record use and associated factors among healthcare professionals at public health facilities in Dire Dawa, eastern Ethiopia: A mixed-method study

**DOI:** 10.3389/fdgth.2022.935945

**Published:** 2022-08-23

**Authors:** Abebe Tolera, Lamessa Oljira, Tariku Dingeta, Admas Abera, Hirbo Shore Roba

**Affiliations:** School of Public Health, College of Health and Medical Sciences, Haramaya University, Harar, Ethiopia

**Keywords:** electronic medical record use, perceived EMR system quality, public health facilities, eastern Ethiopia, perceived service quality

## Abstract

**Background:**

Despite the significant benefits of digital health technologies (ITs), developing countries are lagging behind their developed counterparts in the adoption of electronic medical records (EMRs) in a healthcare setting. EMRs have long been considered essential elements in improving the quality of healthcare. However, the rate of utilization of EMRs among healthcare providers still remains low, particularly in developing countries.

**Objective:**

This study aimed at exploring EMR use and its determinants among healthcare providers at public health facilities in Dire Dawa, eastern Ethiopia.

**Methods:**

A quantitative cross-sectional study was conducted among 402 health professionals working at public health facilities supplemented with an exploratory qualitative study in Dire Dawa, Ethiopia. Descriptive summary statistics and binary and multivariable logistic regression analysis were used to explore the determinant factors of EMR use, while qualitative data were thematically analyzed.

**Results:**

Overall, about a quarter (26.6%) of health professionals were using electronic medical records. A work experience of 6 years or less [adjusted odds ratio (AOR) = 2.23; 95% confidence interval (CI): [1.15–4.31]], a discussion on EMR (AOR = 14.47; 95% CI: [5.58–7.57]), the presence of an EMR manual (AOR = 3.10; 95% CI: [1.28–7.38]), and a positive attitude toward the EMR system (AOR = 11.15; 95% CI: [4.90–25.36]) and service quality (AOR = 8.02; 95% CI: [4.09–15.72]) were independent determinants of EMR use. Poor collaboration among stakeholders and dependence on the software programs of NGOs were the main challenges cited by key informants.

**Conclusion:**

The findings of this study indicate that EMR use by health professionals in the study area is very low. Several organizational, technical, and behavioral factors were identified for this low utilization. Therefore, there is a need to leverage EMRs through continuous technical support and commitment to enhance its use, which has the potential to improve health service performance. Developing locally applicable EMR software should be considered.

## Introduction

The ever-increasing integration of highly diversified technologies in the healthcare field has resulted in the need for gathering organized and accurate data for informed decision-making in the health sector (1–4). Evidence shows that health facilities with electronic notes, records, test results, and clinical decision support lead to an effective overall healthcare delivery system (5–9). One of the record number of automations being implemented in a healthcare setting is the electronic medical record (EMR), which is the “legal record created in hospitals and ambulatory environments that enables health facilities to capture, store, analyze and communicate patient health information in an electronic format” (10, 11).

Despite large investments to support the adoption of EMRs, the adoption rate of such systems is still low, and little progress has been made to harness the benefits of EMRs, particularly in developing countries (12). The implementation of the EMR is not consistent across healthcare facilities and its use is met with an alarming rate of failure in resource-poor settings (13–15). Due to limited resources, most electronic systems are still being used side by side with paper documentation, which is creating a burden on health service providers (16–18).

In developed countries such as the USA, more than three-quarter of health professionals (81.7%) are using EMRs, while in developing countries like Ethiopia, the use of EMR by health professionals is very low (19, 20). A study in sub-Saharan countries showed “the complexity and impact of social considerations, outweighing product and EMR system limitations” (21). Similarly, the utilization of digital health investments for planning and decision-making in Ethiopia has not been a priority so far and generally inadequately supported and poorly managed (3).

The current health sector transformation plan (HSTP) of Ethiopia envisioned “all of its citizens enjoying equitable and affordable access to all types of health services” through the transformation agenda of the Information Revolution (3). In this regard, the use of the electronic medical record represents one of the key instruments in improving healthcare delivery (6, 7, 22). The Ethiopian “Ministry of Health with support of the Tulane University Technical Assistance Project in Ethiopia (TUTAPE) and CDC started the development and implementation of a comprehensive EMR system for hospitals called SmartCare in 2009. The system was deployed in 5 hospitals in Addis Ababa and other hospitals in regional cities. In 2013, the Ministry of Health adapted the system as a national EMR for all hospitals, and planned to scale it up to further hospitals and regions” (23, 24). All urban health facilitates in the Diredawa Administration were selected for the initial phase of implementation and chosen as a pilot site (23).

However, the current rate of utilization since EMR implementation in Dire Dawa is unknown (24). Moreover, no study has been conducted on the utilization status of EMRs and individual and organization determinants in the study area. Therefore, this study is aimed to fill the knowledge gaps related to EMR utilization and its determinants among healthcare providers in the Dire Dawa city administration in eastern Ethiopia.

## Methods

### Study design and setting

An institution-based quantitative cross-sectional study supplemented with an exploratory qualitative approach was conducted at public health facilities in the Dire Dawa city administration from April 7 to May 7, 2019. Dire Dawa, an industrial hub and home to several market centers, is located in eastern Ethiopia and has 14 health centers, 2 hospitals, and 32 health posts with a total of 629 health professionals including health extension workers.

### Study participants

The participants for this study were all healthcare providers selected randomly across two hospitals and seven health centers. A sample size of 446 was estimated with an assumption of a 95% confidence level, 80% power, and 10% non-response rate. However, as the numbers of healthcare providers were manageable (489), all healthcare providers were included in the data collection process. To better understand what factors influence EMR use, we also conducted in-depth interviews with nine key informants who had better knowledge and experience of the EMR in their organizations. The key informants were purposefully selected on the basis of their role in Health Management Information System (HMIS) and healthcare data quality issues. All healthcare providers who were selected randomly across health facilities and who served for at least 6 months were approached to fill up the questionnaire.

### Data collection and quality control methods

A pretested semistructured questionnaire was used, which was supplemented with in-depth interview guides. These tools were adapted and constructed from PRISM tools (25) and from previously conducted studies (24, 26, 27). Data were divided into two types, namely, quantitative data and qualitative data; five clinical nurses and five health officers were employed for collecting quantitative data, whereas two data collectors experienced with qualitative data collection carried out the in-depth interviews. Healthcare providers who were unavailable at the time of study were repeatedly visited to minimize the rate of high non-responses. Three item questions with in-depth probing were adopted and contextualized from other studies (28, 29). The key informant interviews were sound-recorded using the Sony ICD PX470 sound recorder. To avoid desirability bias, none of the study participants were made to know about the data collectors in person. Upon completion of each in-depth interview, a trained biostatistician produced a complete transcript and translation. Strict supervision and double data entry were made for data quality control.

### Measurements

*The dependent variable:* EMR use was measured if a participant used the EMR for one or more of the following functions: (1) finding patients with certain characteristics, (2) making notes (history and physical exam), (3) entering orders (lab, radiology), (4) reviewing/obtaining lab and radiology results, (5) updating diagnosis, (6) reviewing currently received medications, (7) writing prescriptions, (8) admitting a patient, (9) referring a patient, (10) viewing/scheduling appointment for a patient, (11) communicating using Smart Care’s communication/report sending, and (12) producing patient summary reports/report generating. The respondent's computer skills were assessed using a 10-question score as follows: Internet browsing, calculations, email communication, database management, ability to check data accuracy, plot data by months or years, compute trends from bar charts, explain findings and their implications, use data for identifying gaps and setting targets, and use data for making various types of decisions and providing feedback. A mean score >95 denoted excellent, 80–95, very good, 65–80 good, 50–64 fair, and <50 poor. *Perceived EMR system quality* was assessed using five-item questions (please check the Questionnaire in Supplementary Materials), and a two-scale score was used to classify it as good or poor. *Perceived service quality* was assessed using nine-item questions (please check the Questionnaire in supplementary materials), and a two-scale score was used to classify it as good or poor. Similarly, *perceived information quality* was assessed using seven-item questions (please check the Questionnaire in Supplementary Materials), and a two-scale score was used to classify it as good or poor.

### Data analysis

All analyses were performed using STATA 14.1 version. Descriptive summary statistics were used to describe the characteristics of study participants with EMR use. Bivariate and multivariable logistic regression was employed to identify factors affecting EMR use. All statistics with a *p*-value <0.05 were declared significant. The collected qualitative data were transcribed, coded, and thematically analyzed using ATLAS ti 7.5.4. Coding was made inductively during data analysis. The inductive approach was used by semantically analyzing the explicit content of the data to determine our themes.

### Ethical considerations

This study was done following the principles stated in the Declaration of Helsinki. This study was approved by the Institutional Health Research Ethics Review Committee of Haramaya University with Reference number IHRERC/104/2019. Permission for data collection was obtained from health facilities. Written and signed informed consent was obtained from the study participants in a form provided with the Questionnaire.

## Results

### Socio-demographic characteristics of study participants

Out of the 489 study participants, 402 (82.2%) responded to the questionnaires. The majority (55%) of the respondents were females (Table 1). The mean age of the respondents was 31.30 (±6.61 SD) years. More than one-third of the participants (37.8%) had more than 6 years of experience, whereas about three-quarter (76.6%) of the respondents were degree holders. For the qualitative in-depth interviews, seven HMIS/health informatics technicians (HIT) staff members and two health facility heads were involved. Five of the key informants were females. All of them were married; six of them were degree holders.

**Table 1:** Socio-demographic characteristics of the study participants, Dire Dawa, Ethiopia, 2019 (*n* = 402).

Variable	Category	Freq.	Percent
Sex	Female	219	54.48
	Male	183	45.52
Age (years)	20–24	36	8.96
	25–29	151	37.56
	30–34	123	30.60
	35 and above	92	22.89
Residence	Urban	342	85.07
	Rural	60	14.93
Marital status	Single	160	39.80
	Married	231	57.46
	Others	11	2.74
Education	Diploma	77	11.16
	Degree	308	76.62
	Masters and above	17	4.23
Work experience	≤ 6 years	250	62.19
	>6 years	152	37.81
Profession	All Nurses	256	63.68
	Laboratory technicians	45	11.2
	Physicians	38	9.46
	Health Officers	32	7.96
	Pharmacist	31	7.71
Average monthly Income in ETB	601–1,650	10	2.49
	1,651–3,200	50	12.44
	3,201–5,250	186	46.27
	5,251–7,800	97	24.13
	7,801–10,900	43	10.70
	Over 10900	16	3.98

ETB, Ethiopian birr.

### Accessibility, functional status, and computers skills of health professionals

A majority of the study participants 243 (60.45%) had access to at least one computer in their working desk (excluding the personal computer), of which about 85% were functional at the time of the study. (Table 2) More than two-third of the current users (178 (73.25%)) are using this computer (s) for data recording, and an additional 141 (58.02%) and 74 (30.45%) of them use the available computer (s) for report generating and reading, respectively.

**Table 2:** Accessibility, functional status, and computer skills of healthcare professionals, Dire Dawa (*n* = 402).

Variable	Categories	Frequency	Percent
Have access to computer(s)	Yes	243	60.45
	No	159	39.55
Number of accessible computer(s)	1	178	73.25
	2	42	17.28
	3	11	4.53
	≥4	12	4.94
Computer's functionality	Yes	207	85.19
	No	36	14.81
Share the available computer(s) with others	Yes	194	79.84
	No	49	20.16
Number of health professional(s) with whom they share their computers	0	47	19.34
	1	19	7.82
	2	38	15.64
	3	51	20.99
	4 and above	88	36.21
Computer skill	Fair	202	50.25
	Poor	200	49.75

### EMR use and related characteristics

**Figure 1:**
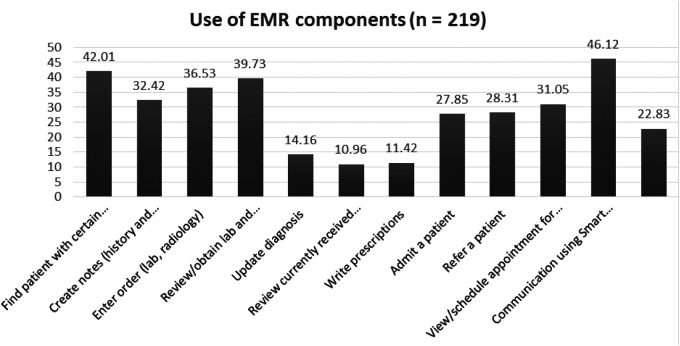
Use of the basic EMR components by health professionals, Dire Dawa, Ethiopia, 2019.

Overall, 388 (96.52%) of the participants are aware of the EMR and more than half (54.48%) of them have used it before. Just over a quarter of health professionals (107 (26.61%) are currently using EMRs. The EMR is most commonly used for sending reports (46.12%), followed by finding patients with certain characteristics 42.01% (Figure 1).

**Figure 2:**
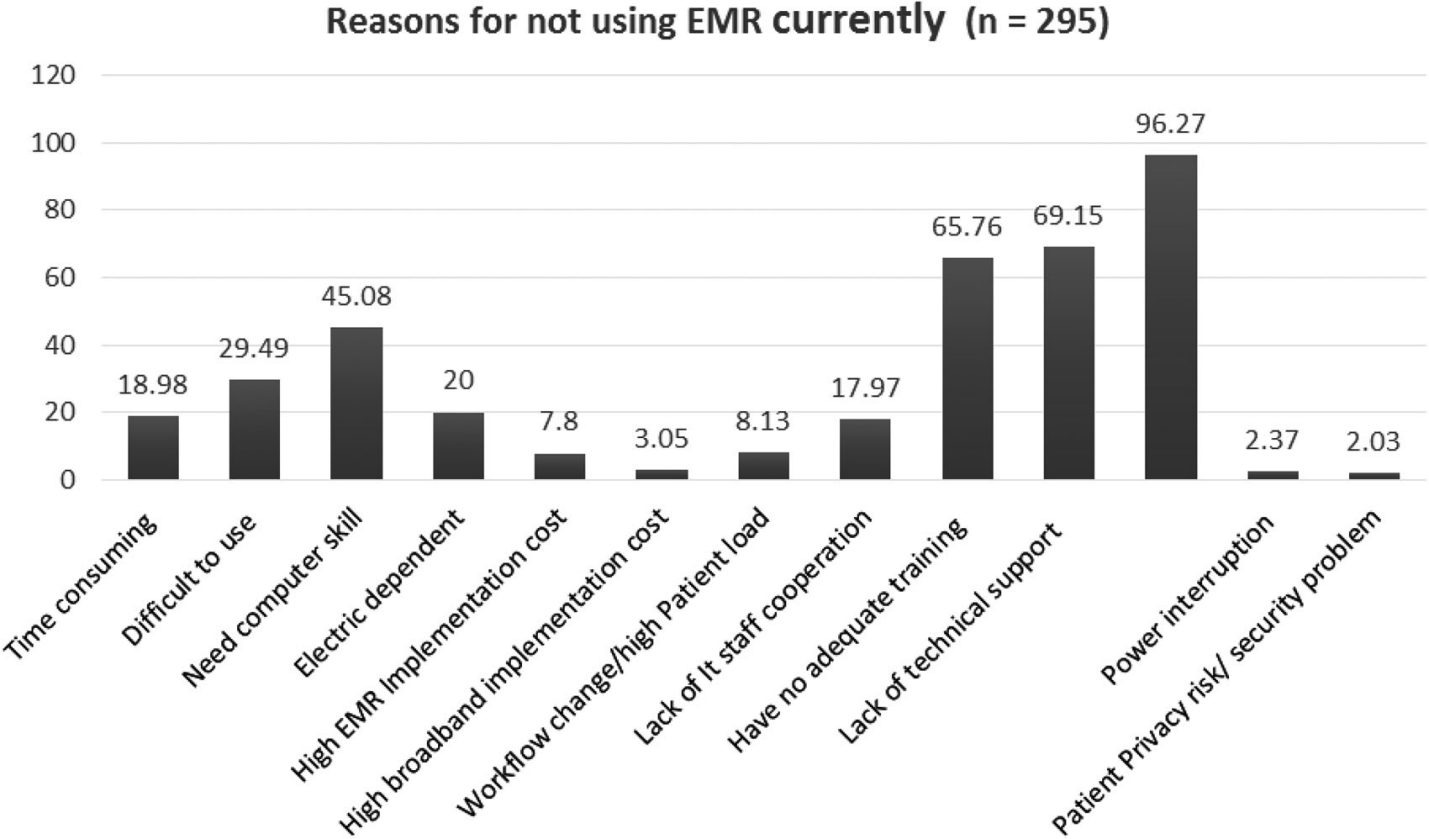
Reported reasons for not currently using the EMR system Dire Dawa, Ethiopia, 2019.

Furthermore, about two-thirds of the current users 71 (66.4%) are nurses, followed by laboratory technicians 15 (14.02%), health officers 10 (9.35%), physicians 10 (9.35%), and pharmacists. Moreover, the main reported reasons for the current non-use of the EMR are the unavailability of the functional installed EMR software program [the SmartCare installed by Tulane University is outdated due to project phase-out (Figure 2)].

The majority 260 (64.68%) of the participants have received HMIS training, while 267 (66.42%) have been trained on EMR use. However, only 174 (65.17%) of the participants use EMRs after training, and only 63 (15.67%) of the participants own the EMR manual. Furthermore, only 33 (8.21%) participants hold regular discussions on the EMR during performance monitoring team meetings.

### Health professionals' acceptance and attitude toward the EMR

**Figure 3:**
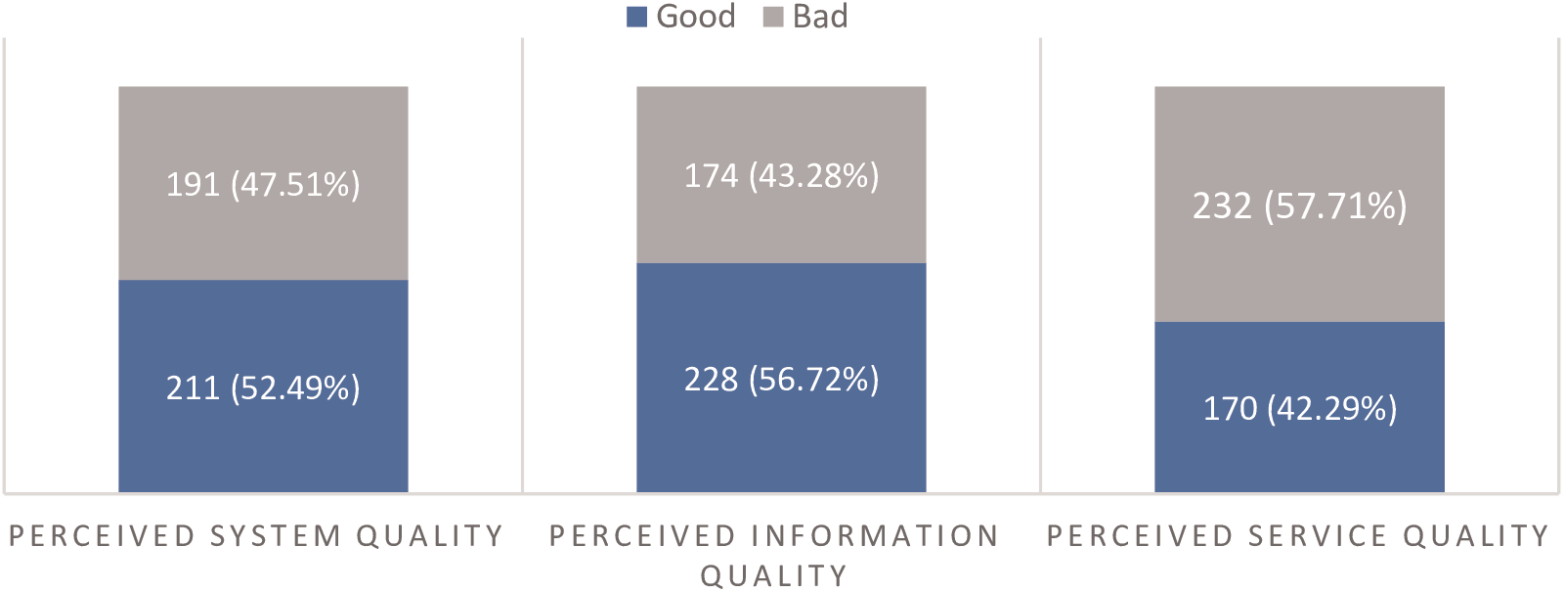
Study participants’ perceived EMR system, information, and EMR service quality, Dire Dawa, Ethiopia, 2019.

In general, when respondents were asked about EMRs, 287 (71.39%), 256 (63.68%), 255 (63.43%), and 266 (66.17%) of them agreed that they fully accepted them, believed that they improved their productivity, preferred them over paper-based record, and agreed that electronic recording of patient data had an impact on data quality, respectively. Moreover, there were significant differences in EMR use among healthcare providers with respect to their views on EMR service quality, EMR system quality, and perceived EMR information quality (Figure 3).

### Factors associated with EMR use

In the final multivariable logistic regression analysis, the following variables were found to independently predict EMR use; work experience, access to the EMR manual, discussions on the EMR in meetings, positive perceived EMR system quality, perceived service quality, and perceived benefit of the EMR (Table 3).

**Table 3:** Factors associated with EMR use by health professionals working in urban public health facilities, Dire Dawa, Ethiopia, 2019 (*n* = 402).

Variables	Response	EMR use (%)		COR (95% CI)	AOR (95% CI)	*p*-Value
		Use	Do not use			
Work experience	>6 years	31 (20.4)	121 (79.6)	1	1	
	≤6 years	76 (30.4)	174 (69.6)	1.70 (1.06–2.75)	2.13 (1.08–4.20)	0.03
Computer skill	Poor	40 (20.0)	160 (80.0)	1	1	
	Fair	67 (33.2)	135 (66.8)	1.98 (1.26–3.12)	1.32 (0.68–2.56)	0.41
Smart care training	No	26 (19.3)	109 (80.7)	1	1	
	Yes	81 (30.3)	186 (69.7)	1.82 (1.10–3.01)	0.45 (0.20–1.01)	0.06
Presence of EMR manual	No	66 (19.5)	273 (80.5)	1	1	
	Yes	41 (65.1)	22 (34.9)	7.71 (4.30–13.82)	3.01 (1.23–7.40)	0.02
Having discussions on EMRs in any meeting	No	62 (17.9)	284 (82.1)	1	1	
	Yes	45 (80.4)	11 (19.6)	18.74 (9.2–38.3)	15.23(5.70–40.74)	0.001
Perceived service quality	Poor	23 (9.9)	209 (90.1)	1	1	
	Good	84 (49.4)	86 (50.6)	8.88 (5.25–15.00)	8.31 (4.11–16.80)	0.001
Perceived information quality	Poor	25 (14.4)	149 (85.6)	1	1	
	Good	82 (36.0)	146 (64.0)	3.35 (2.02–5.53)	1.87 (0.85–4.08)	0.12
Perceived system quality	Poor	13 (6.8)	178 (93.2)	1	1	
	Good	94 (44.6)	117 (55.4)	11.00 (5.9–20.55)	7.38 (2.97–18.34)	0.001
Perceived EMR benefits to facilities	Do not benefit	67 (23.4)	219 (76.6)	1	1	
	Benefit	40 (34.5)	76 (65.5)	1.72 (1.07–2.75)	0.33 (0.06–1.65	0.18
Perceived EMR benefits to patients	Do not benefit	65 (22.0)	231 (78.0)	1	1	
	Benefit	42 (39.6)	64 (60.4)	2.33 (1.45–3.76)	5.51 (1.10–27.67)	0.04

EMR, electronic medical record; AOR, adjusted odds ratio; CI, confidence intervals: COR, crude odds ratio.

Respondents with a work experience of 6 years or less were about two times more likely to use EMRs than those with a work experience of greater than 6 years [adjusted odds ratio (AOR) = 2.13; 95% confidence interval (CI) = [1.08–4.20]]. Study participants who had access to the EMR manual were three times more likely to use the EMR system than those who had no EMR manual (AOR = 3.01; 95% CI = [1.23–7.40]). Those health professionals who reported having a discussion on EMRs in any meeting were about 15 times more likely to use them compared with those who did not have any discussion on them (AOR = 15.23; 95% CI = [5.70–40.74]). Respondents with good perceived EMR service quality were eight times more likely to use EMRs than those with a poor perception of EMR service quality (AOR = 8.31; 95% CI = [4.11–16.80]). Similarly, respondents with good EMR system quality were about seven times more likely to use EMRs than those with poor perceived EMR system quality (AOR = 7.38; 95% CI [2.97–18.34]).

Health professionals who thought EMRs will benefit patients were about 5.5 times more likely to use them than those who said they will not benefit patients [AOR: 5.51; 95%CI (1.10–27.67)] (Table 3).

### Challenges of EMR use (qualitative finding)

Multiple factors that influence EMR use were cited by key informants during the in-depth interviews. Common themes were organized as organizational, technical, and behavioral factors during analysis (Table 4). The most common significant barrier cited by key informants for not using EMRs was the lack of EMR software installed on computers as well as lack of management commitment to integrate EMRs into patient data recording.

**Table 4:** Common themes identified during in-depth interviews on factors affecting health professionals’ EMR use.

Organizational factors	sGood governance problems, (ii) lack of budget for training and maintenance, (iii) dual documentation system, (iv) poor supervision and support, (v) work overload/shortage of time, (vi) inadequate HIT professionals, (vii) lack of incentives for good performance, (viii) poor coordination and mentoring process, (ix) ownership problems from health institutions.
Technical factors	(i) lack of a locally designed EMR software program, (ii) old and non-functional computers, (iii) electricity interruption, (iv) Inadequate EMR training, (v) lack of timely maintenance and repair of computers, (vi) expired EMR software program.
Behavioral factors	(i) lack of computer skills and knowledge, (ii) poor commitment from users and management, (iii) lack of interest in adapting to a computerized system, (iv) challenges in motivating staff, (v) carelessness from staff and management, (vi) intentional resistance to use EMRs.
Environmental factors	(i) Poor collaboration from stakeholders, (ii) dependence on NGOs’ software program, (iii) hot weather conditions.

HMIS focal person from the Dire Dawa City Health Bureau reported: “Poor commitment from all health professionals and governing body working in the facilities takes the major share of barriers. Some health professionals are not even interested and motivated to manually record patient data. Some health professionals also consider working with computer as an extra burden.” This finding is supplemented by the quantitative finding where despite receiving training on EMR use, most health professionals were not using it.

Another point raised by key informants is health professionals’ attitude toward the EMR (Table 4). Some respondents cited that some health professionals get agitated with filling data in computers and rather prefer to use paper-based record.

Technical factors were also cited as one factor for the non-use of EMRs*.* A 32-year-old HIT staff at one of the health facilities said, “If the program malfunctioned or gets corrupted, there was no timely maintenance.” He added, “There was no replacement for even a single malfunctioned Socket or divider.”

## Discussion

This study revealed that only a quarter of health professionals were using EMRs. This finding indicated low use compared with a finding in the USA (13), Hyder hospital in Tigray, and Amhara states in Ethiopia (24, 30, 31). None of the urban health facilities had a fully functional EMR system. A few service delivery units such as antiretroviral treatment (ART) clinics and patient registration offices were using the EMR system in conjunction with paper-based records. This resulted from technical challenges related to EMRs, non-functional computers, electricity interruption, and the lack of timely maintenance and repair of computers. Moreover, healthcare providers’ behavioral factors such as poor computer literacy, poor commitment, and lack of interest in adapting to electronic recording were mentioned.

This study also found that poor collaboration from stakeholders and dependence on NGOs for the EMR program were cited as major factors. The respondents indicated that the software was designed by a non-governmental organization (NGO) (Tulane University), and after the project was phased out, the EMR service was also stopped due to the lack of necessary infrastructure for integrating the EMR with other existing information systems. Other studies indicated that the dependence of developing countries on third-party vendor systems for EMRs, which usually involve unsustainable IT infrastructure and software management, were identified as significant barriers in EMR implementation (23, 32–34).

However, this study finding is in line with those of other studies conducted in Ethiopia's Amhara Region (30) and in Addis Ababa (24). The lack of administrative and policy support and lack of available funding are cited by key informants as major barriers to EMR use. More than two-third of the respondents are in favor of EMR implementation and its positive effects on quality of care, which is supported by studies conducted in the Tigray region (27) and USA (35). The literature indicates that most health professionals have accepted the role of automated notes and records in improving healthcare quality (5, 36), though the lack of infrastructure has hindered the full implementation of the system. In general, barriers associated with hardware and software resources are the most commonly cited barriers to EMR use by health professionals in this study.

Health professionals with a work experience of 6 years or less are twice more likely to use EMRs compared with those with work experience greater than 6 years. This finding is similar to that of a study conducted in northern Ethiopia (27). Studies have shown that the gap in the knowledge and skills of health workers significantly influences data management processes (3, 37). Variations in terms of computer literacy, educational level, and personal commitment in this study could be an explanation for low EMR use among health professionals. Having discussions on EMRs in any meeting and the presence of the EMR manual are predictors of EMR use in this study. These findings are in line with those of other studies elsewhere in Ethiopia (20, 24, 27) and the USA (38).

In contrast to studies conducted in different regions of Ethiopia (24, 27, 37), Iran (39), the USA (38), and Cyprus (40), training in the EMR system had no influence on EMR use in our study. From the logical point of view, training can improve the knowledge, attitude, and skills of health professionals. However, having adequate training by itself cannot be a driving factor unless the EMR system is continuously maintained and the staff are motivated to use EMR. In addition, key informants stated that the benefits of training have not reached those who actively engage in EMR use, including card room and ART units. Inadequate IT professionals have also been cited by key informants as a reason for low EMR utilization.

Further, this study revealed that the attitude toward EMR system quality influences the utilization of the EMR system. Health professionals with good opinion on EMR service quality and EMR system quality are more likely to use EMRs than their counterparts are. This finding is consistent with that of a study conducted in Addis Ababa (24). This is evident as users' acceptance is the primary determinant of effective utilization of any program (41, 42).

The qualitative finding identified major organizational, technical, and behavioral factors as the major obstacles to EMR use by health professionals. These determinants have also been cited by other studies from the USA (43), Saudi Arabia (28), and the UK (29). Organizational (13, 35, 39, 44), technical, and most importantly behavioral (38, 39) factors were identified to be important determinants hindering effective and efficient utilization of EMRs.

This cross-sectional study included only urban public health facilities. An extensive overview of overall EMR utilization status requires an inclusion of health posts and rural and private health facilities. The generalizability of the finding of this study may not apply to these facilities. Further, use was not assessed from a multilevel perspective across individual and organizational levels.

## Conclusions

The immense benefits offered by EMRs were very poorly utilized in this study area. Only a quarter of the respondents surveyed said that they used EMRs in their day-to-day activities. Organizational determinants (EMR manual presence, absence of EMR software program, smart care training, and attending regular discussions on the EMR) and individual factors (views on the benefits of the EMR to patients, perceived system quality, and service quality) had a significant influence on the utilization of the EMR. Successful utilization of the EMR requires the support and commitment of all stakeholders. Interventions should focus on improving user support, stabilizing power fluctuations, improving computer infrastructure, and providing continuous training.

## Data Availability

The original contributions presented in the study are included in the article/Supplementary Material, further inquiries can be directed to the corresponding author.
