# Supplementary material for: Electronic medical record use and associated factors among healthcare professionals at public health facilities in Dire Dawa, eastern Ethiopia: A mixed-method study
Source: Front Digit Health. 2022 Aug 23;4:935945. doi: 10.3389/fdgth.2022.935945 (PMC9697183; doi:10.3389/fdgth.2022.935945)
Supplement: Supplementary file 1 [file Table5.doc]

Haramaya University

Harar

Haramaya.edu.et

**Dr. Björn Wolfgang Schuller (Professor, PhD).**

**Field Chief Editor**

**Frontiers in Digital Health**

**May 4, 2022**

**Dear Dr. Björn Wolfgang Schuller**

We wish to submit our original research article entitled “**Electronic Medical Record Use and Associated Factors among Healthcare Professionals at Public Health Facilities in Diredawa, Eastern Ethiopia: A mixed Method Study”** for consideration by Frontiers in **Digital Health.**

We confirm that this work is original and has not been published elsewhere, nor is it currently under any consideration for publication elsewhere.

In this paper we found that EMR utilization by health care providers in urban public health facilities in Dire Dawa is very low (only one quarter of health professionals were using EMR). This is significant because use of electronic medical record represents one of the key instruments in improving the efficiency, effectiveness, timeliness, quality, and safety of healthcare delivery. Without appropriate use of the ever-increasing technologies in health care fields, availing organized, accessible, timely, and accurate data for informed decision making become difficult which may hinder improvement in health of the community. Given low utilization of EMR, we believe the findings presented in our paper will appeal to local program planners and specific scientific community who subscribe to **Frontiers in Digital Health**

Please address all correspondence concerning this manuscript to [komiintolera@gmail.com](mailto:komiintolera@gmail.com)

Thank you for your consideration of this manuscript

Sincerely

Mr. Abebe Tolera (MPH in Epidemiology).
